# Regenerative tissue filler for breast conserving surgery and other soft tissue restoration and reconstruction needs

**DOI:** 10.1038/s41598-021-81771-x

**Published:** 2021-02-01

**Authors:** Theodore J. Puls, Carla S. Fisher, Abigail Cox, Jeannie M. Plantenga, Emma L. McBride, Jennifer L. Anderson, Craig J. Goergen, Melissa Bible, Tracy Moller, Sherry L. Voytik-Harbin

**Affiliations:** 1 GeniPhys, LLC, Zionsville, IN 46077 USA; 2 Division of Surgery, Indiana University School of Medicine, Indianapolis, IN 46202 USA; 3 Department of Comparative Pathobiology, College of Veterinary Medicine, Purdue University, West Lafayette, IN 47907 USA; 4 Department of Veterinary Clinical Sciences, College of Veterinary Medicine, Purdue University, West Lafayette, IN 47907 USA; 5 Weldon School of Biomedical Engineering, College of Engineering, Purdue University, West Lafayette, IN 47907 USA; 6 Medical Scientist/Engineer Training Program, Indiana University School of Medicine, Indianapolis, IN 46202 USA; 7 Department of Basic Medical Sciences, College of Veterinary Medicine, Purdue University, West Lafayette, IN 47907 USA

**Keywords:** Surgical oncology, Preclinical research, Translational research, Biomaterials, Regenerative medicine, Tissue engineering

## Abstract

Complete removal of cancerous tissue and preservation of breast cosmesis with a single breast conserving surgery (BCS) is essential for surgeons. New and better options would allow them to more consistently achieve this goal and expand the number of women that receive this preferred therapy, while minimizing the need for re-excision and revision procedures or more aggressive surgical approaches (i.e., mastectomy). We have developed and evaluated a regenerative tissue filler that is applied as a liquid to defects during BCS prior to transitioning to a fibrillar collagen scaffold with soft tissue consistency. Using a porcine simulated BCS model, the collagen filler was shown to induce a regenerative healing response, characterized by rapid cellularization, vascularization, and progressive breast tissue neogenesis, including adipose tissue and mammary glands and ducts. Unlike conventional biomaterials, no foreign body response or inflammatory-mediated “active” biodegradation was observed. The collagen filler also did not compromise simulated surgical re-excision, radiography, or ultrasonography procedures, features that are important for clinical translation. When post-BCS radiation was applied, the collagen filler and its associated tissue response were largely similar to non-irradiated conditions; however, as expected, healing was modestly slower. This in situ scaffold-forming collagen is easy to apply, conforms to patient-specific defects, and regenerates complex soft tissues in the absence of inflammation. It has significant translational potential as the first regenerative tissue filler for BCS as well as other soft tissue restoration and reconstruction needs.

## Introduction

Breast cancer is the most commonly diagnosed cancer in women, with over 2 million new cases every year world-wide and approximately 330,000 per year in the United States alone^1,2^. It is estimated that 60–70% of cases per year (~ 1.3 million globally) are treated with breast conserving surgery (BCS; otherwise known as lumpectomy). BCS involves removal of the tumor along with a cancer-free margin of healthy tissue (negative margins), preferably through a small, cosmetically placed incision. BCS with adjunct radiation is preferred over mastectomy (i.e., removal of the whole breast) for eligible patients, because it yields equivalent survival while preserving patients’ breasts and reducing surgery time, recovery time, and complications^3–5^. Since 5- and 10-year survival rates for women with breast cancer are relatively high, greater than 90%^2^, long-term outcomes and survivorship are especially important when treating this disease. Specifically, for BCS, complete removal of cancerous tissue and preservation of breast shape, appearance, and consistency (i.e., pleasing breast cosmesis) in a single surgery are paramount to achieving satisfactory outcomes and patient quality of life.

According to the American Society of Breast Surgeons, standard guidelines for BCS involve “closing the surgical defect in layers as cosmetically as possible” following resection of the tumor^6^. Healing of the complex surgical wound follows, initially with a seroma or hematoma forming in the defect, followed by scar formation and contraction. For surgeons, it is extremely challenging, if not impossible, to predict the cosmetic outcome of BCS, especially given significant patient variation in breast tumor size, shape, and location, and the unpredictable nature of the tissue repair process, which is compounded by the effects of adjunct radiation therapy. Because of this, there remains a relatively high level of BCS-related breast deformities, with approximately one-third of women developing dents, distortions, and asymmetry between breasts^7,8^. Such outcomes are known to negatively impact the self-esteem, body image, and intimacy of breast cancer survivors, contributing to overall feelings of insecurity, anxiety, and depression^9,10^. Furthermore, the need for secondary surgical procedures, which increases healthcare costs, remains high for BCS, with estimates ranging from 20 to 40%^11,12^. This includes re-excisions due to positive margins as well as revision and reconstruction procedures to repair breast deformities. Due to these concerns, BCS may not be an ideal option for all women, especially those with tumors that are large in comparison to breast size (> 5 cm in diameter; tumor:breast volume percent greater than 1.5%) or positioned within the lower quadrants of the breast^13–15^. Therefore, breast surgeons are in need of new options to further optimize oncologic and cosmetic outcomes of BCS, enabling them to confidently offer this conservative therapy to more patients with satisfying outcomes.

At present, there are no commercial products that allow surgeons to predictably restore, reconstruct, or regenerate soft tissues, such as the breast. Furthermore, it is apparent that breast surgeons are actively looking for solutions to this problem. For example, BioZorb represents a relatively new, three-dimensional, spiral-shaped tumor bed marker intended to mark the surgical cavity for targeted post-operative radiation. However, breast surgeons have used this bioresorbable device with hopes that it would also assist in filling the tissue void and improving cosmetic results. Published clinical studies indicate that both surgeons and patients have been uniformly dissatisfied with BioZorb since this implant is relatively expensive, does not significantly improve outcomes, and gives rise to a hard, palpable lump that lasts for up to 2.8 years and causes patient discomfort^16,17^.

On the other hand, there are two surgical reconstruction options which aim to improve BCS cosmetic outcomes, namely autologous fat grafting (also known as lipofilling or fat transfer) and oncoplastic surgery^18,19^. Fat grafting involves harvesting fat (adipose tissue) via liposuction from one area of the patient’s body and re-injecting minimally processed fat cells into another region. Originally fat grafting was used for delayed breast reconstruction procedures, but more recently it has been investigated for use immediately following BCS. While this approach has seen some success, persistent challenges remain, including rapid reabsorption leading to significant volume loss (ranging from 25 to 80%), fat necrosis, oil cyst formation, micro-calcifications, and questions around oncologic safety (i.e., cancer recurrence)^20–23^. As an alternative approach, oncoplastic surgery combines the skills of surgical oncology with the techniques of plastic surgery to reconstruct one or both breasts at the time of lumpectomy. Oncoplastic procedures include both volume displacement (rearrangement of remaining healthy breast tissue) and volume replacement (reconstruction with various autologous tissue flaps) techniques. While oncoplastic surgery offers the advantage of using the patient’s own tissue, this approach is limited by need for specialized training, involvement of multiple surgeons, longer surgical procedures, and increased cost^18,24^.

In the present study, we aimed to develop and evaluate a soft tissue filler that would (1) predictably restore and regenerate damaged tissue and tissue voids, (2) be easily applied, (3) conform to patient-specific defects varying broadly in size and geometry, and (4) not interfere or compromise routine clinical processes and procedures. In particular, type I oligomeric collagen (oligomer), a highly-purified molecular form of collagen that is readily soluble in dilute acid^25,26^, represents a tunable, in situ forming biomaterial with potential to address many of these design considerations. Unlike conventional monomeric collagen preparations, namely telocollagen and atelocollagen, oligomer represents small aggregates of full-length triple-helical collagen molecules (i.e., tropocollagen) with carboxy- and amino-terminal telopeptide intact, held together by a naturally-occurring intermolecular crosslink. The preservation of these key molecular features provides this natural polymer with desirable but uncommon properties. More specifically, oligomer retains its fibril-forming (self-assembly) capacity, which is inherent to fibrillar collagen proteins and yields scaffolds which recreate the structural and biological signaling features of collagen scaffolds found in the extracellular matrix (ECM) component of tissues^25,26^. Further, upon neutralization to physiologic conditions (e.g., pH and ionic strength), oligomer solutions can be readily applied to fill complex contours and geometries, where the liquid rapidly transitions to a fibrillar collagen scaffold^27–29^. Upon in vivo implantation, these scaffolds persist, showing slow metabolic turnover and remodeling, resistance to proteolytic degradation, and no active biodegradation or foreign body response^27–33^. Finally, oligomer supports creation of materials with broadly tunable physical properties, including geometry, architecture (random or aligned fibrils, continuous fibril density gradients), and mechanical integrity^30,31,33–37^, making it an enabling platform for personalized regenerative medicine.

Here, we evaluated prototype oligomer formulations specifically designed to serve as a regenerative filler for damaged or defective soft tissues, such as the tissue void created by BCS. First, prototype in situ forming collagen scaffolds were characterized based on molecular purity, polymerization (self-assembly) time, and viscoelastic properties. To evaluate biocompatibility and effectiveness of these scaffolds, simulated lumpectomy procedures were performed on the breasts (mammary glands) of pigs. Prototype formulations were used to fill a subset of lumpectomy voids, and surgical outcomes were compared to untreated defects (no fill; negative control) and normal breasts on which no surgery was performed (positive controls). To define the tissue response timeline and gain insight into oligomer mechanism of action, a 16-week longitudinal study was performed. Additionally, a second study was conducted to assess how the collagen scaffold and its associated tissue response was affected by post-operative irradiation. Outcome measures included semi-quantitative visual and palpation-based examination, ultrasonography, radiography, and gross and histological analyses. Combined, these data provide preclinical support for the use of this regenerative tissue filler during breast conserving surgery.

## Results

### Liquid collagen conforms to geometry and transitions to stable, fibrillar scaffold with properties similar to soft tissues

**Figure 1:**
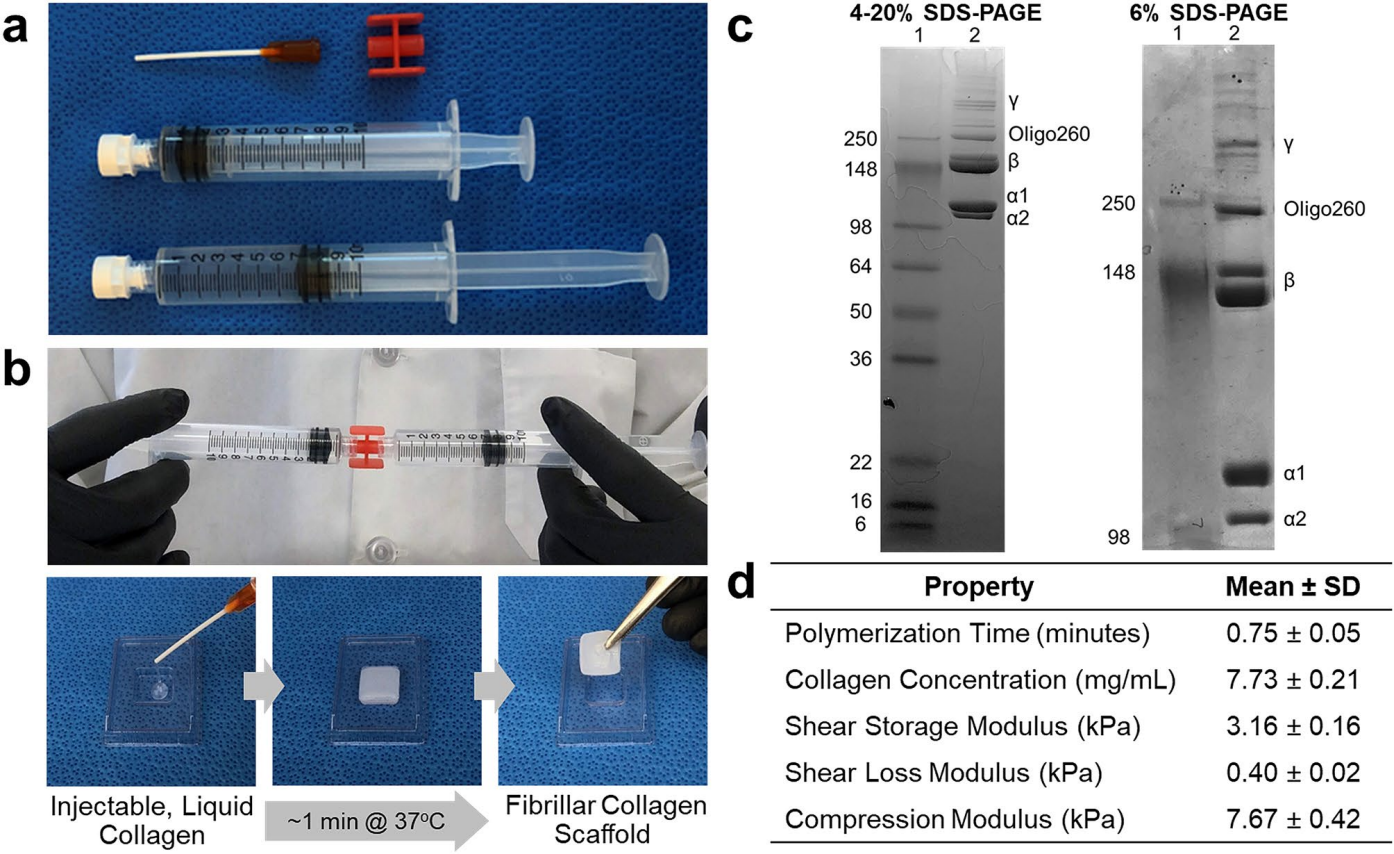
Purified liquid collagen forms viscoelastic fibrillar scaffold with soft tissue-like properties. (**a**) Kit consisting of syringe containing sterile type I oligomeric collagen solution, a syringe of propriety neutralization (self-assembly) buffer, a luer-lock adapter, and applicator tip. (**b**) Images showing mixing of two reagents followed by injection into a plastic mold maintained at body temperature (37 °C), where the liquid transitions into a stable, shape-retaining fibrillar collagen scaffold. (**c**) SDS–PAGE (4–20% and 6% gels) documenting purity and characteristic banding pattern of type I oligomeric collagen. Images represent full length gels and show all relevant lanes. Lane 1: molecular weight standard. Lane 2: type I oligomeric collagen. Uncropped images of the full gel length are shown in Supplementary Figure S1. (**d**) Table summarizing collagen polymerization kinetics and performance specifications (mean ± SD; N = 4, n = 6–8) of prototype collagen scaffold.

Prototype scaffold-forming collagen formulations were obtained as kits from GeniPhys (Zionsville, Indiana). As shown in Fig. 1a, the kit consisted of a syringe containing sterile type I oligomeric collagen in dilute acid (0.01 N hydrochloric acid), a syringe containing a sterile proprietary neutralization solution, a sterile luer-lock adapter, and a sterile applicator tip. Immediately prior to use, the two syringes were joined with the luer-lock adapter (Fig. 1b) and the collagen and neutralization reagent mixed at a ratio of 9 to 1, bringing the collagen solution to physiologic pH and ionic strength. After mixing, the viscous liquid could be injected into various geometries, where it conformed to the shape prior to transitioning into a physically-stable, fibrillar collagen scaffold (Fig. 1b, Supplementary Video S1). To demonstrate collagen purity, sodium dodecyl sulfate polyacrylamide gel electrophoresis (SDS–PAGE) was performed using 4–20% and 6% gels. Gels revealed a banding pattern characteristic of oligomeric collagen^25^ with no detectable contaminating non-collagenous proteins or other types of collagens (Fig. 1c, Supplementary Fig. S1). Other functional performance parameters, including polymerization time and viscoelastic properties of formed collagen scaffolds, were measured, with a summary provided in Fig. 1d. Specifically, the concentration of oligomer prior to neutralization was roughly 7.7 mg/mL. Upon neutralization, the scaffold-forming reaction took, on average, just under 1 min, as measured rheometrically at 37 °C. When analyzed in oscillatory shear and unconfined compression, the formed scaffold exhibited solid-like behavior with shear storage (G′) and loss (G″) moduli of 3.16 ± 0.16 kPa and 0.40 ± 0.02 kPa, respectively, and a compressive modulus of 7.67 ± 0.42 kPa. Although scaffold properties are tunable across a broad range of elastic modulus and strength values, the formulations tested here were designed to exhibit viscoelastic properties similar to soft tissues.

### Collagen scaffold maintains volume; induces vascularization and breast tissue regeneration with no inflammation

**Figure 2:**
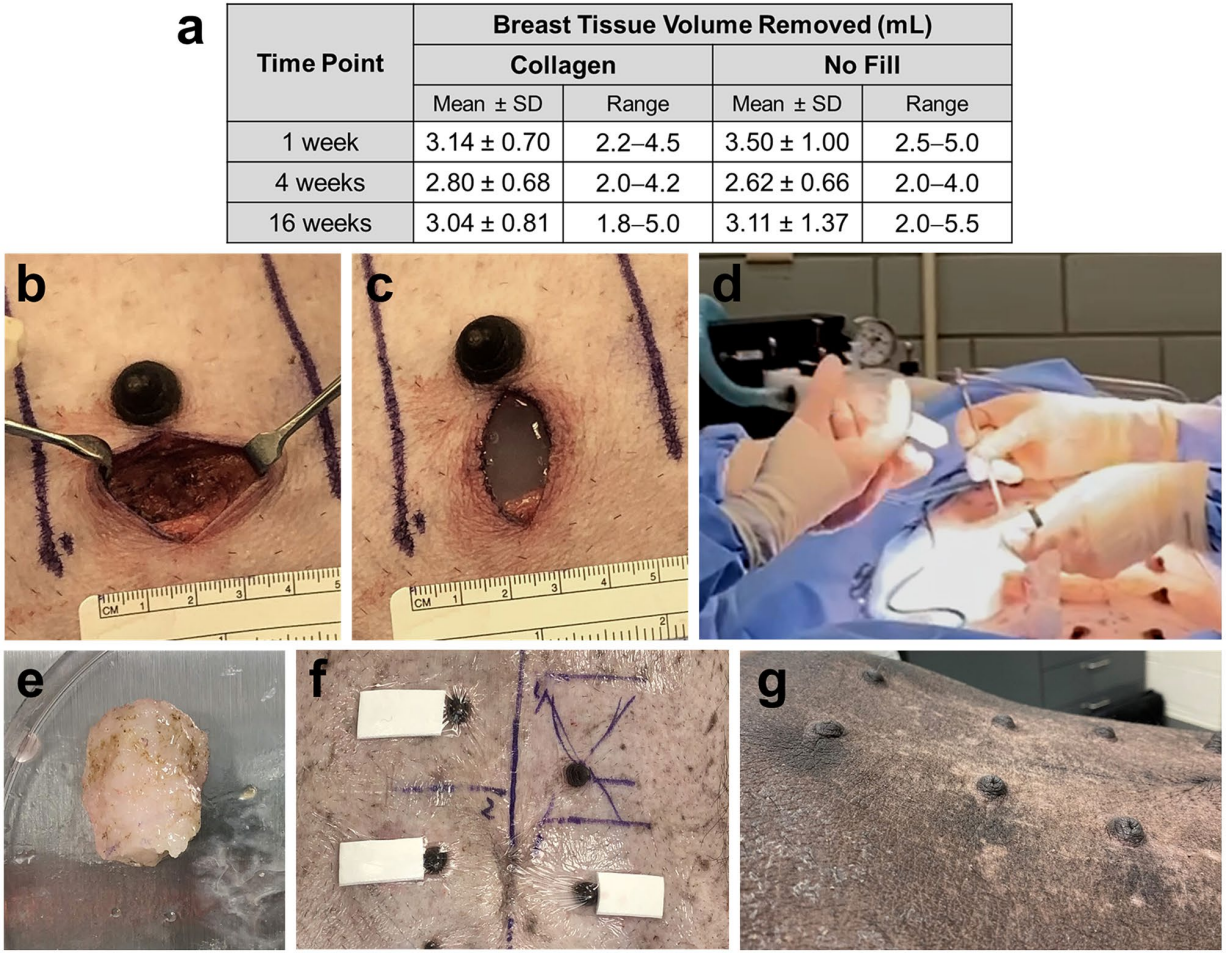
Overview of simulated lumpectomy procedure. (**a**) Table summarizing surgically excised mammary tissue volume, which represented roughly one-fourth total breast tissue volume. Data (mean ± SD) compiled from both longitudinal and radiation studies (1 week: collagen filler: n = 12, no fill = 6; 4 weeks: collagen filler: n = 18, no fill: n = 9; 16 weeks: collagen filler: n = 18, no fill: n = 9). Surgical void (**b**) before and (**c**) after filling with collagen. (**d**) Application of scaffold-forming collagen. (**e**) Excised mammary tissue. Surgical sites (**f**) immediately following surgery showing bandaging and (**g**) 16 weeks following simulated lumpectomy with irradiation.

To evaluate the effectiveness of the scaffold-forming collagen as a regenerative filler for soft tissue defects, a longitudinal study was performed involving simulated lumpectomy procedures on breasts of normal, healthy Yucatan mini-pigs (Fig. 2). Female mini-pigs represent the preferred large animal model for such translational studies based on their size and anatomical and physiological similarities to humans^38^. Additionally, pigs generally have twelve mammary glands (breasts), which reduced the total number of animals required for the studies since each breast could serve as an experimental or control group. Roughly one quarter of breast tissue volume was excised (Fig. 2e), which ranged from 2 to 5.5 mL of tissue (average ~ 3 mL) depending upon individual breast size (Fig. 2a). For collagen-treated breasts, the liquid collagen was mixed and immediately injected into the tissue void, where it conformed to the complex geometry prior to transitioning to a fibrillar collagen scaffold in less than 5 min under these circumstances (Fig. 2b–d). The breast surgeon used her discretion when filling each defect, with applied collagen volumes varying with defect size and geometry. Surgical voids were filled with at least the same volume of collagen as tissue removed, with the majority receiving 1–2 mL more collagen volume. Negative control sites were left untreated (no fill), which is consistent with standard-of-care BCS procedures. All incisions were closed using resorbable sutures and bandaged (Fig. 2f). All animals maintained weight (± 5 kg), surgical sites remained closed, and no procedural complications occurred throughout the duration of the study (Fig. 2g).

Consistent with what is observed amongst women and men, pig breasts were found to vary in volume, consistency, and composition both within and between individual animals. At the microscopic level (Supplementary Fig. S2), mammary glands consisted of multiple lobes, composed of smaller secretory lobules organized as clusters and a system of ducts (channels) that eventually exited the skin via the nipple. The lobules and ducts were supported by an intralobular stroma, composed predominantly of fibrous type I collagen. Additionally, collagenous connective tissue was found between lobes (interlobular stroma), providing support to the breast and determining its shape. Adipose tissue, which primarily determines breast size, filled the space between the glandular and fibrous connective tissue. When evaluated in unconfined compression, breasts located cranially (toward the head) were relatively stiff, with an average compression modulus of 19.0 ± 12.9 kPa. Progressing caudally (toward the tail), breasts increased in fat composition and were softer, with an average compression modulus of 6.56 ± 2.51 kPa for the most caudal breasts.

**Figure 3:**
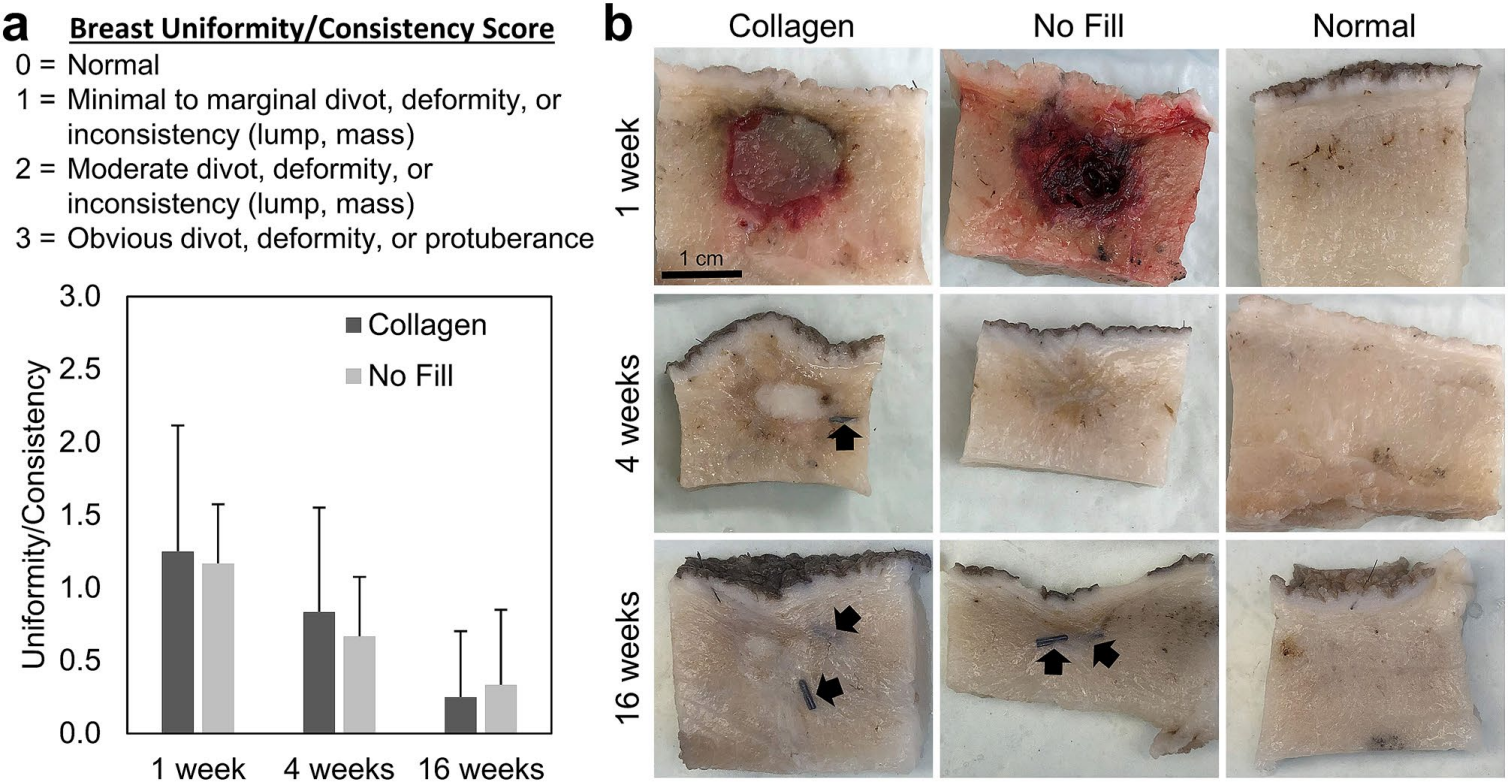
Collagen filler persists and induces site-appropriate tissue regeneration. (**a**) Graph showing breast uniformity/consistency scores (mean ± SD; collagen: n = 12; no fill: n = 6) assigned by breast surgeon for collagen and no fill (negative control) treated voids at various time points following simulated lumpectomy. All no surgery breasts scored 0. (**b**) Cross-sections of surgical voids following treatment with collagen or no fill compared to normal breast tissue. Arrows represent surgical clips placed to mark boundaries of surgical void.

To assess biocompatibility and tissue response of the collagen filler, animals were anesthetized at designated time points of 1, 4, and 16 weeks. All breasts were examined visually, palpated, and semi-quantitatively scored in a blinded fashion according to criteria in Supplementary Fig. S3. Collagen-treated and no fill control breasts showed no evidence of erythema (redness) or eschar (sloughing, dead tissue) at any time point. Mild edema was evident at 1 week in breasts on which surgery was performed; however, the extent of swelling was similar for both collagen and no fill groups and subsided shortly thereafter. Uniformity/consistency scores for collagen-treated breasts were similar to no fill controls at all three time points, decreasing from roughly 1.2 at 1 week to 0.25 by 16 weeks (Fig. 3a). Such findings are important because they indicate that the collagen filler does not create breast inconsistencies that could be interpreted clinically as residual disease or a source of patient discomfort. All normal breasts received a score of zero. Additionally, when the breast surgeon performed simulated surgical re-excision on collagen-treated breasts, the fill material did not compromise or interfere with the procedure.

**Figure 4:**
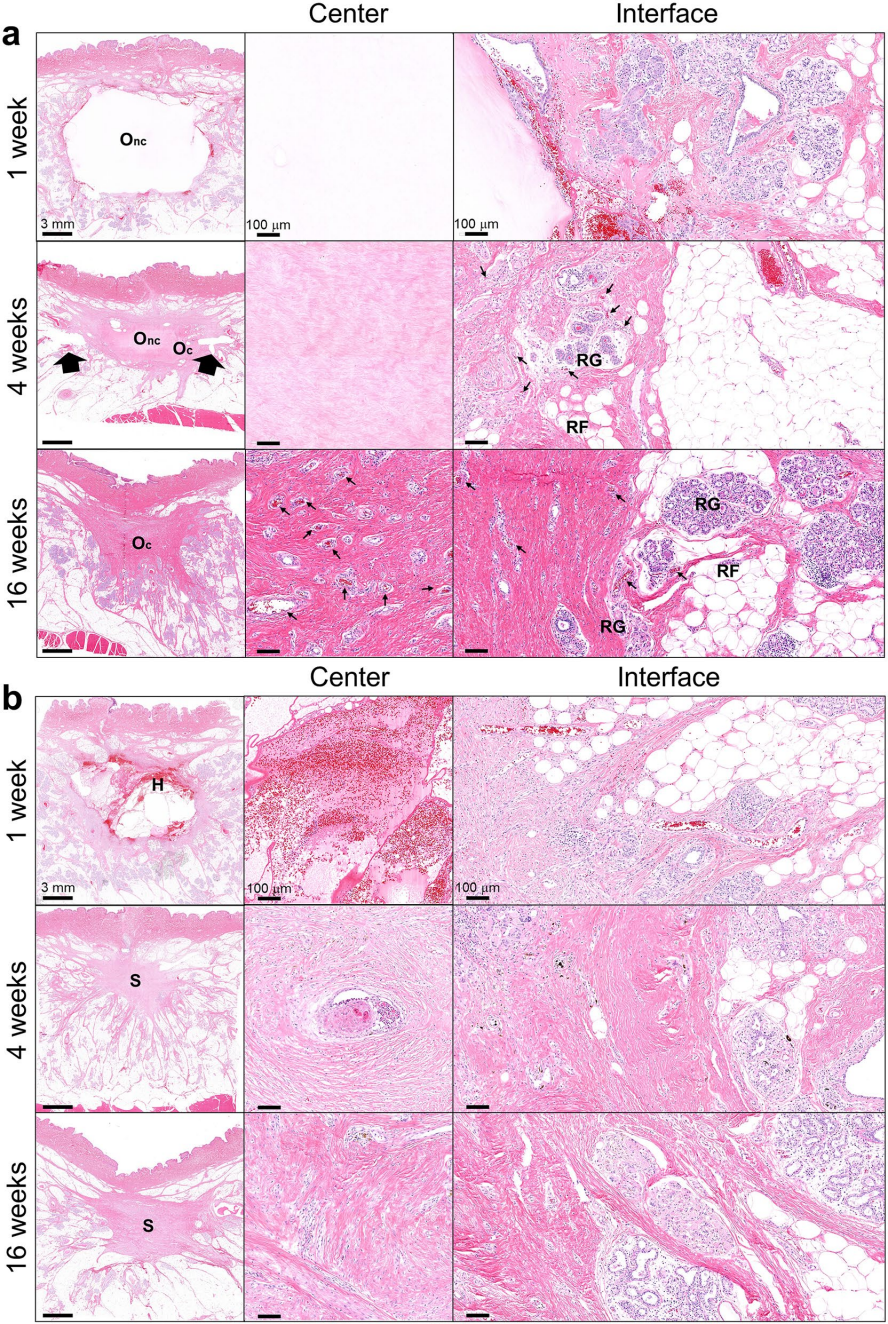
Collagen filler supports breast tissue neogenesis without evoking an inflammatory response. (**a**) Cross-sections (H&E) of collagen filled voids at 1 week, 4 weeks, and 16 weeks following simulated lumpectomy. Low magnification images show treated voids and their interface with the surrounding host tissue (large arrows indicate surgical clip sites). High magnification images feature the central region of the collagen filler and the filler/host tissue interface. Cellular infiltration, vascularization, and site-appropriate tissue generation of the scaffold occur over time. By 16 weeks, the collagen is completely cellularized and vascularized (small arrows indicated blood vessels) with evidence of regenerated mammary gland (RG) and adipose tissue (RF). O_nc_: oligomer scaffold with no cell infiltration, O_c_: oligomer scaffold with cellular infiltrate. (**b**) Cross-sections (H&E) of untreated (no fill) surgical voids at 1 week, 4 weeks, and 16 weeks following simulated lumpectomy. Low magnification images show voids and the surrounding host tissue. High magnification images feature the central region of the voids and the void/host tissue interface. Hematomas (H) were commonplace at 1 week, followed by progressive defect contraction and scar tissue formation (S).

Biocompatibility and tissue response of the collagen filler were further defined based on gross and histological examination of transverse sections of breast explants, with comparisons to no fill and normal breast controls. From these analyses, it was apparent that the collagen filler maintained its volume (minimized defect contraction), was highly biocompatible, and exhibited a regenerative tissue response in absence of an inflammatory reaction or foreign body response. As cells infiltrated the scaffold and new breast tissue was generated, it took on a tissue-like appearance that was difficult to discern grossly from surrounding normal tissue (Fig. 3b). In this case, the surgical clips were useful as markers of the original defect margins (Figs. 3b, 4). Upon histological analysis at 1 week, the collagen filler was evident within the tissue void, where it appeared as a homogenous, light pink (eosinophilic) staining material (Fig. 4a). Often surrounding the filler was a band of hemorrhage, fibrin, and a few leukocytes, which was attributable to the surgical manipulation of the tissue (Fig. 4a). At the filler-host tissue interface, there were focally extensive areas of proliferating fibroblasts (mesenchymal cells) with few small-caliber vessels infiltrating the scaffold edges. The surrounding breast tissue appeared largely normal, with remodeling areas adjacent to the surgical site. These regions contained aggregates of remodeling epithelial cells, some of which appeared to be ductules while others were more irregularly shaped, suggestive of rudimentary lobules (Fig. 4a). It is noteworthy that there was no evidence of an inflammatory-mediated foreign body reaction or active biodegradation that is characteristic of conventional implantable materials^39^. At the 4-week time point, fibroblasts, along with newly formed vasculature, extended into deeper portions of the collagen filler, with infiltrating cells most abundant at the periphery and dwindling further into the center (Fig. 4a). Multifocal aggregates of epithelial cells were observed, which were again consistent with precursors of glandular structures (Fig. 4a). By 16 weeks, the scaffold was completely cellularized, appearing as mature, remodeled collagen fibers and bundles, with some sites displaying small discernible regions of acellular eosinophilic filler material. Small caliber vessels were present diffusely and evenly distributed throughout the scaffold (Fig. 4a). Within the vascularized collagen scaffold, adipose tissue and cytokeratin-positive lobules and ducts were present, especially at the periphery (Fig. 4a, Supplementary Fig. S4). The glandular morphology was well developed and mature with no remarkable pathology.

By contrast, at 1 week, hematoma formation was evident upon both gross and histological evaluation of no fill breast explants (Figs. 3b, 4b). Hemorrhage, fibrin clot, and leukocytes were evident within the lumpectomy site. Intermixed within areas of hemorrhage were proliferating fibroblasts with few small caliber vessels, consistent with fibrovascular tissue associated with reparative wound healing. Scattered necrotic regions with active inflammation were also apparent surrounding the defect area. By 4 weeks, these tissue defects contracted as evidenced by significant clip displacement grossly and a star-like, constricted appearance histologically (Fig. 4b). Fibrovascular scar tissue was prominent within the defect area, with multiple, small regions of necrosis and inflammation noted throughout and near the defect border (Fig. 4b). Active remodeling of glandular and adipose tissue was observed in tissue regions surrounding the defect (Fig. 4b). By 16 weeks, the fibrous scar tissue increased in density, appearing as differentially oriented swirls of parallel-aligned fibrous tissue densely populated by myofibroblasts. While lobules, ducts, and adipose tissue were identified surrounding the defect, multiple glands with poorly developed morphological features were found within the scar tissue periphery, as evidenced by the presence of inflammatory cells and low-level, diffuse cytokeratin staining. (Fig. 4b, Supplementary Fig. S4).

### Collagen scaffold does not compromise interpretation of sonograms and radiographs

**Figure 5:**
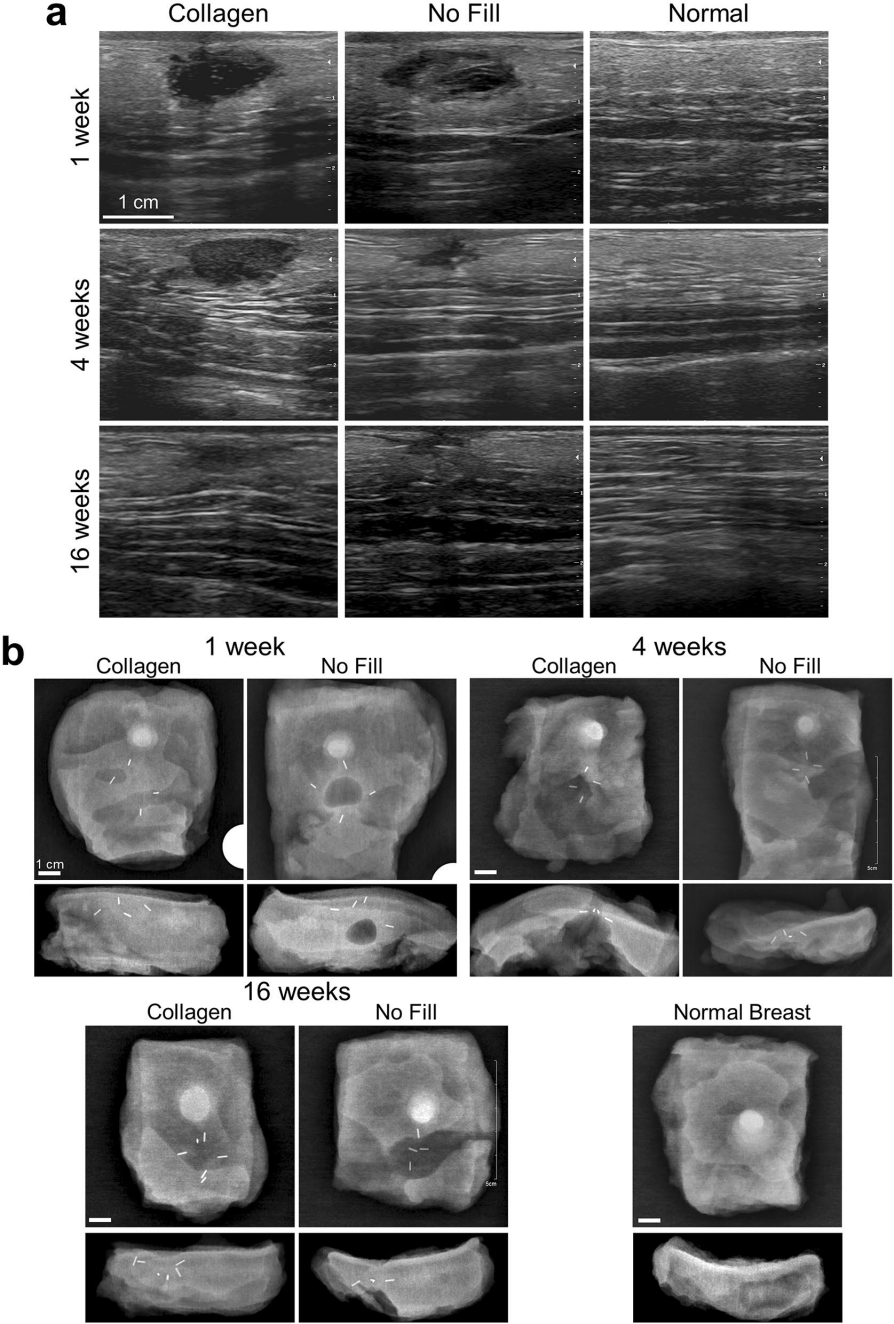
Collagen filler does not interfere with radiography or ultrasonography. Representative (**a**) ultrasound images and (**b**) radiographs of surgical voids treated with collagen or no fill compared to normal breast tissue at 1-week, 4-week, and 16-week time points. Radiopaque marker clips evident within radiographs indicate boundaries of surgical void and show evidence of contraction for no fill voids.

Mammography and ultrasonography are routinely used as follow-up diagnostic procedures to BCS to monitor for cancer recurrence. To ensure that the collagen filler did not compromise or interfere with image interpretation, ultrasound was performed on all pig breasts prior to euthanasia and radiographs were taken of each individual whole breast following mastectomy. Sonograms obtained over the 16-week study showed that the collagen scaffold did not obscure or prevent interrogation of breast tissue and did not produce any regions of unexpected echogenicity (Fig. 5a). At 1 week, a large, irregularly-shaped hypoechoic region was observed within collagen-treated breasts containing varying degrees of heterogeneous echoes (Fig. 5a). Such signals were not surprising given that the filler microstructure represents a randomly-oriented meshwork of collagen fibrils measuring roughly 400 μm in diameter^25^. While these regions appeared to maintain their volume over time, they gradually took on the appearance of normal tissue, which corroborated the cellularization and vascularization observed within gross explants and histologically (Fig. 5a). No fill treated voids also showed an irregular-shaped hypoechoic region consistent with seroma and hemorrhage at 1 week (Fig. 5a). By 4- and 16-week time points, these regions diminished in size, producing a heterogeneous signal consistent with contraction and scar formation (Fig. 5a).

The collagen scaffold also did not interfere with radiograph interpretation, but rather displayed an opacity consistent with normal tissue throughout the duration of the study (Fig. 5b). Additionally, radiographs provided further evidence that the collagen scaffold maintained the void volume with limited clip displacement over time (Fig. 5b). The majority of untreated (no fill) surgical voids also produced radiographs that appeared consistent with normal tissue at 1 week, with a small number of sites displaying obvious darkened regions consistent with an air pocket, seroma, or hematoma (Fig. 5b). The progressive displacement of surgical clips observed at 4- and 16-week time points provided further evidence of defect contraction and scarring over time (Fig. 5b).

### Irradiation does not adversely affect collagen filler or regenerative response

To determine if the collagen filler was compatible with radiation therapy, a cohort of animals was subjected to ventral irradiation two weeks following the simulated lumpectomy procedure, with each animal receiving a total dose of 20 Gy over 5 consecutive days. Irradiated animals displayed an increase in skin pigmentation over time as evidenced by a darkening of skin color (Fig. 2g), which would be expected in humans undergoing therapeutic irradiation as well. At the microscopic level, moderate hyperplasia or thickening of the epidermis was evident with increased melanin deposition especially within the basal epidermis (Supplementary Fig. S2). At 16 weeks, breast tissue was noticeably stiffer, again a common change observed with radiation therapy^40^. Additionally, signs of fat necrosis and atypical hyperplasia of ducts and glands were evident (Supplementary Fig. S2)^41^.

**Figure 6:**
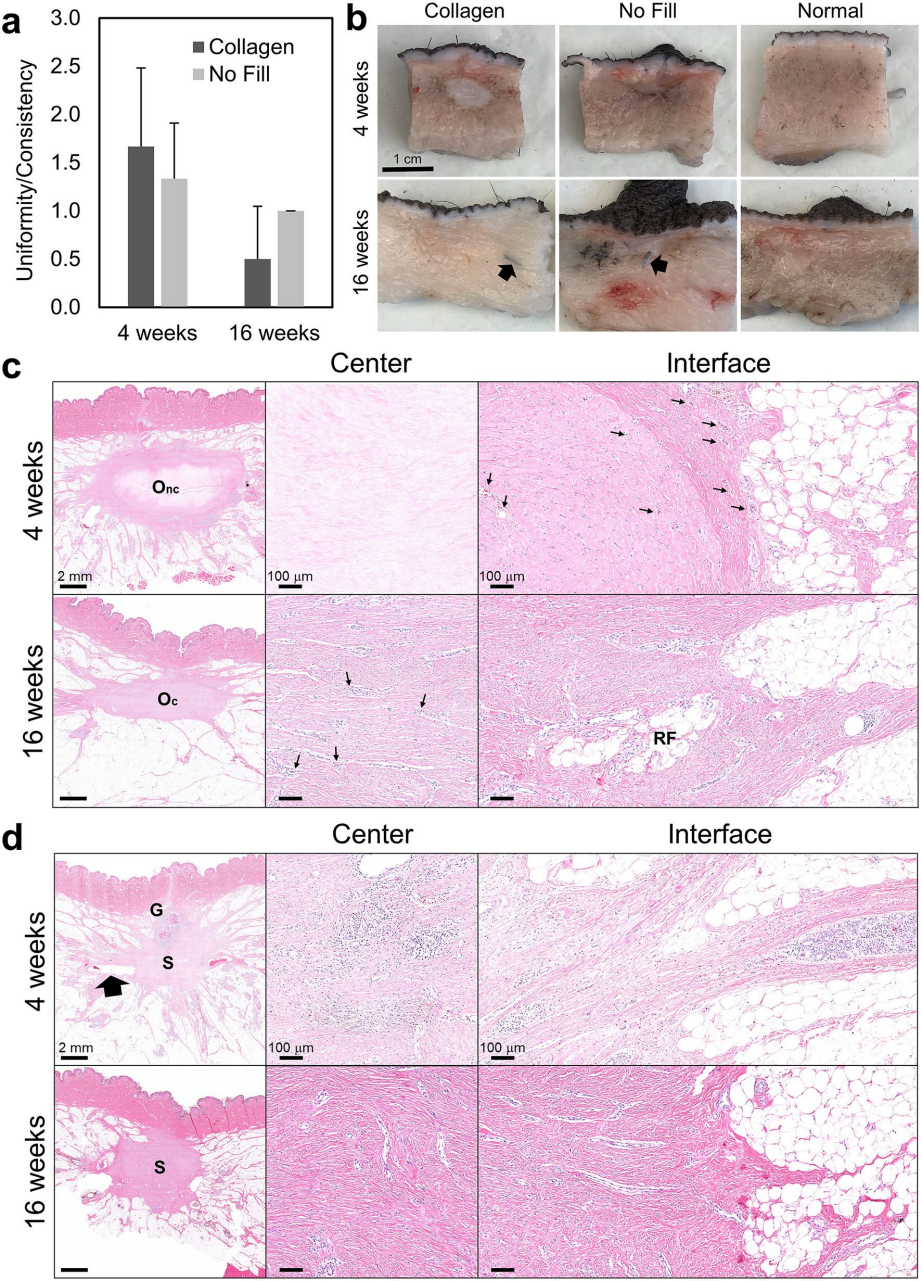
Radiation has little to no effect on collagen filler and associated tissue response. (**a**) Graph showing breast uniformity/consistency scores (mean ± SD; collagen: n = 6; no fill: n = 3) assigned by breast surgeon for collagen treated and no fill (negative control) voids at various time points following simulated lumpectomy and radiation. All no surgery breasts scored 0. (**b**) Cross-sections of surgical voids following treatment with collagen or no fill and radiation compared to no surgery normal breast tissue. Arrows represent surgical clips placed to mark boundaries of surgical void. (**c**) Cross-sections (H&E) of collagen filled voids at 4 weeks and 16 weeks following simulated lumpectomy with irradiation. Low magnification images show treated voids and their interface with the surrounding host tissue. High magnification images feature the central region of the collagen filler and the filler/host tissue interface. Cellular infiltration, vascularization, and site-appropriate tissue generation of the collagen implant occur over time, albeit at a slower rate than sites from non-irradiated animals. By 16 weeks, the collagen is completely cellularized and vascularized (small arrows indicated blood vessels) with evidence of regenerated adipose tissue (RF). O_nc_: oligomer scaffold with no cell infiltration, O_c_: oligomer scaffold with cellular infiltrate. (**d**) Cross-sections (H&E) of untreated (no fill) surgical voids at 4 weeks and 16 weeks following simulated lumpectomy with radiation. Low magnification images show voids and the surrounding host tissue, with scar tissue (S) and a suture-related granuloma (G) evident at 4 weeks (large arrow indicates surgical clip site). High magnification images feature the central region of the scar tissue and the scar/host tissue interface.

**Figure 7:**
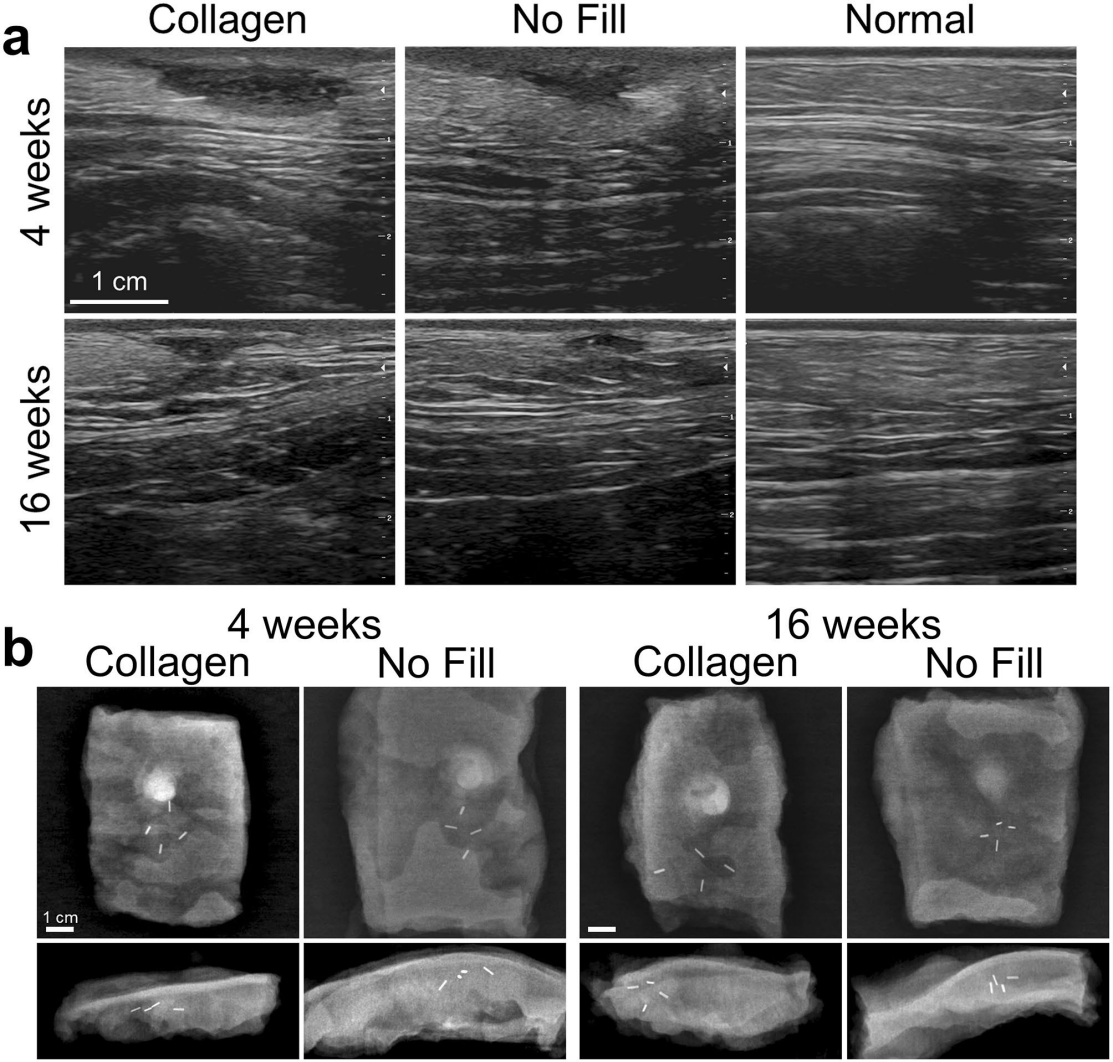
Collagen filler does not compromise interpretation of diagnostic images of breast tissue even after irradiation. Representative (**a**) ultrasound images and (**b**) radiographs of surgical voids treated with collagen or no fill and irradiation compared to normal breasts at 4-week and 16-week time points. Radiopaque marker clips evident within radiographs indicate boundaries of surgical void and show evidence of contraction for no fill voids.

With the exception of differences in skin pigmentation, all breasts and surgical sites healed well, appearing similar to those of non-irradiated animals. Average breast uniformity/consistency scores for collagen-treated and no fill groups were somewhat higher in irradiated versus non-irradiated animals at the respective time points, with the only exception being the 16-week collagen-treated group, where scores were similar (Figs. 6a, 3a). Examination of gross explants and histological cross-sections revealed no obvious adverse effect of irradiation on the collagen scaffold or its associated tissue response; however, subjectively, the overall healing timeline of irradiated sites appeared modestly delayed (Fig. 6b,c). Over the 16-week study period, the collagen filler persisted within the surgical site, inducing progressive cellularization, vascularization, and breast tissue regeneration, which proceeded inward from the filler-host tissue interface. As expected, the no fill group showed contraction and the development of fibrous scar tissue (Fig. 6b,d). Sonograms (Fig. 7a) and radiographs (Fig. 7b) were largely similar for irradiated and non-irradiated animals, again confirming that the collagen filler was not negatively affected by irradiation and did not produce any suspicious imaging anomalies.

## Discussion

Identifying a therapeutic approach that more predictably and consistently preserves breast shape and appearance following BCS would bring more confidence to both surgeons and patients when selecting conservative therapy such as BCS. Additionally, it would assist in maintaining the quality of life and emotional well-being of millions of breast cancer survivors each year worldwide. Such an approach would also benefit other patient populations in need of soft tissue restoration or reconstruction, including children with congenital defects, individuals suffering from traumatic injuries, and cancer patients requiring resection of tumors within tissues other than breast (e.g., skin, muscle). The mammary glands of miniature swine have been routinely used to evaluate innovative strategies to improve outcomes of breast surgical procedures^42,43^. Here, by performing simulated lumpectomy procedures on female mini-pigs, we show that an in situ forming scaffold formulated from type I oligomeric collagen addresses a number of surgeon and patient needs, with the potential to translate as the first regenerative soft tissue filler. More specifically, our findings show that this specially formulated collagen is easy to use and can be readily applied as part of a surgical procedure (e.g., tumor excision) without disrupting normal workflow. Its liquid format readily fills and conforms to patient-specific defect geometries and contours and is amenable to minimally invasive procedures^27–29^. Once injected into the site, oligomer rapidly transitions to a physically-stable fibrillar collagen scaffold that persists and maintains its volume. As a natural and unmodified fibrillar collagen scaffold, it restores critical biochemical and biomechanical signaling to cells, inducing site-appropriate regeneration of complex tissue compositions, including those found in the breast, in absence of an inflammatory reaction or foreign body response. Finally, we show that the material is compatible with a number of standard clinical procedures, including irradiation, radiography, ultrasonography, and surgical re-excision.

The collagen filler described here is fundamentally different from conventional flowable and injectable collagen products that are or have previously been used for soft tissue augmentation (e.g., cosmetic procedures), management of skin wounds (e.g., ulcers), and tissue bulking (e.g., urinary incontinence). Such products, which include Zyderm, Zyplast, Integra Flowable, and Contigen, are fashioned from reconstituted, enzymatically-treated collagen (atelocollagen) or granulated tissue particulate derived from bovine, porcine, or human tissue sources. To make these materials injectable, the insoluble fibrous collagen or tissue particulate is suspended in physiologic saline solutions to create dispersions or suspensions. All of these implantable collagens are temporary and exhibit rapid biodegradation (reabsorption; 1–6 months), where they are actively degraded via inflammatory-mediated processes, including phagocytosis by macrophages/giant cells and proteolytic degradation by secreted matrix metalloproteinases^44^. To slow degradation and improve persistence, many of these products are treated with glutaraldehyde or other exogenous crosslinking processes^45^.

By contrast, oligomer represents a molecular subdomain found within natural tissue collagen fibers (e.g., porcine dermis), which can be extracted and purified so that it is free from cellular and other immunogenic tissue components. The type I collagen protein and crosslink chemistry comprising this subdomain are highly conserved across species^46^, documenting the significance of this major structural element within the body. Physiologic conditions induce fibril formation, where oligomer molecules assemble into staggered arrays, giving rise to interconnected networks or scaffolds of fibrils^25,26,36^. Published studies show that formed scaffolds are largely similar to those found naturally within the extracellular matrix, comprising fibrils with regular D-banding patterns that readily engage in biosignaling^36^. The natural crosslink chemistry present in oligomer, but not found in polymerizable monomeric collagens, is the primary contributor to the rapid scaffold-forming reaction as well as the improved mechanical integrity, slow metabolic turnover, and resistance to proteolytic degradation exhibited by oligomer scaffolds^25,26,36^. Collectively, these distinguishing features contribute to the uncommon mechanism of action and regenerative tissue response displayed by oligomer scaffolds when compared to conventional biodegradable collagen materials.

The ability to restore and regenerate tissue that is diseased, damaged, or dysfunctional has been one of the greatest challenges in medicine. In fact, researchers have been working to identify biomaterials and/or anti-inflammatory agents with the goal of achieving a more desirable healing outcome (i.e., regeneration) or biomaterial/device implant response^47–50^. For the breast, this challenge is particularly difficult, since it is comprised of multiple tissue types with distinct functions, including secretory (i.e., milk-producing) glands and ducts, supportive collagenous connective tissue, and volume-filling adipose tissue. At present, tissue engineering and regenerative medicine strategies for soft tissue and breast reconstruction remain in their infancy, with only a few strategies evaluated in large animal models to date (for reviews see^49–51^). The majority of approaches have focused on engineering adipose tissue from biologic or synthetic scaffolds, incorporating lipofilling, patient-derived cell populations, and growth factors to encourage adipogenesis and vascularization. For example, Santerre and co-workers developed a porous, biodegradable breast filler from polycarbonate-urethane for BCS applications. In a recent study, scaffold pellets designed with breast-like mechanical properties were used to fill lumpectomy cavities in mini-pigs. These scaffolds showed evidence of primarily an inflammatory cell infiltrate at 6 weeks, with 20–40% scaffold degradation and limited breast tissue regeneration at 9 months^52^. As an alternative approach, Hutmacher and co-workers are applying additive manufacturing techniques to create patient-specific polycaprolactone scaffolds for breast reconstruction. To improve adipose tissue generation, these scaffolds are implanted within the subglandular space of mini-pigs and allowed to accumulate fibrovascular tissue 2 weeks prior to injection of a lipoaspirate^43^. A major drawback to both of these synthetic scaffold approaches is the inability of the materials to signal cells, resulting in foreign body responses and slow cellularization and vascularization^51^.

**Figure 8:**
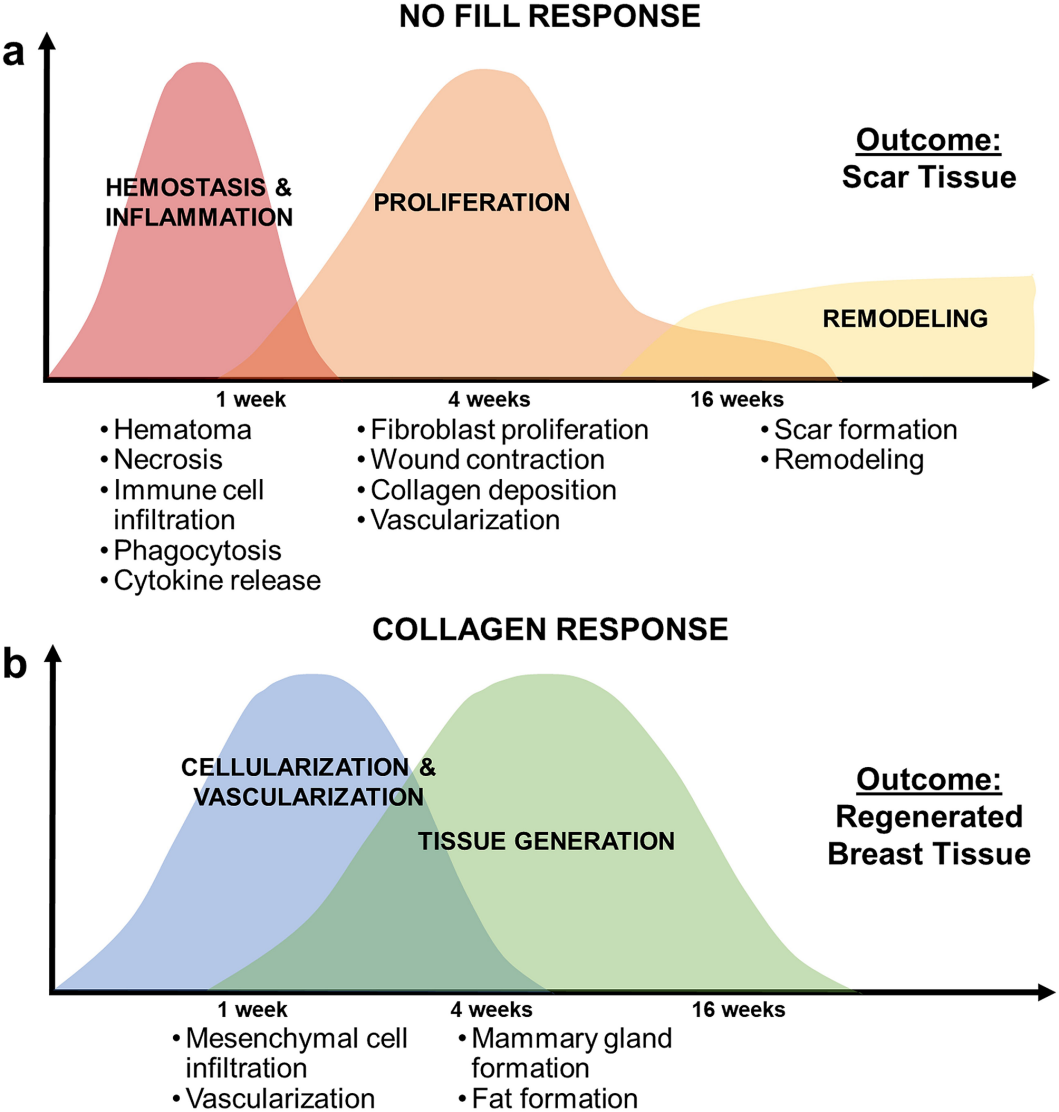
Timelines and processes of healing responses observed in porcine simulated lumpectomy model. Schematics comparing and contrasting the phases and processes associated with the (**a**) normal reparative response observed with no fill and the (**b**) proposed regenerative response observed with the collagen filler.

In this study, porcine breasts varied in size and tissue composition, giving rise to consistency differences that were apparent both qualitatively and quantitatively. The measured compressive modulus range (approximately 6–19 kPa) encompassed breast consistencies observed in women, which reportedly ranges from 0.7 to 66 kPa depending on breast composition (e.g., fibroglandular versus fatty) and testing parameters (e.g., strain rate, preconditioning)^53,54^. The healing response of untreated breast defects was similar to that observed in women following BCS, yielding scar tissue that was structurally and functionally distinct from normal breast tissue. The 16-week longitudinal study showed progression through the classic overlapping phases of reparative wound healing that results in scarring, including hemostasis and inflammation, proliferation, and remodeling as shown in Fig. 8a. Substantial contraction of the defect, as evidenced by clip displacement and star-like scar tissue morphology, was facilitated by the initial fibrin clot and provisional matrix which are mechanically weak compared to normal breast ECM. The process of scar formation and remodeling over time is perhaps the most unpredictable and troubling aspect of BCS, since it is known to contribute to pain, distortions in the breast contour and consistency, and loss of sensation, all of which negatively affect women emotionally and psychologically^55^.

Filling the defect volume with a long-lasting fibrillar collagen, that is naturally metabolized and remodeled rather than actively degraded, resulted in a healing response where immune mediators were largely absent, and the outcome was more regenerative rather than reparative. Based on these results, the proposed regenerative healing response for the collagen filler is depicted in Fig. 8b. Since the injectable scaffold filled and conformed to defects and effectively integrated with surrounding host tissue, it re-established a structural and mechanical continuum across the tissue, which is known to be important to scar-free healing and tissue morphogenesis^56,57^. Notably, the compression modulus (7.67 ± 0.42 kPa) of the collagen filler fell within the range of both pig and human breast mechanical properties. The dense microstructure and compression properties of the collagen filler effectively resisted contraction forces exerted by the surrounding normal tissue as well as infiltrating cells. Additionally, since scaffold mechanical properties were similar to soft tissues, they did not yield any concerning palpable breast inconsistencies. From a translational perspective, this is important for patient satisfaction and comfort, as well as for maintaining the ability to detect recurrent cancer through palpation.

Because collagen fibrils contain multiple functional cellular and molecular binding domains^58^, the scaffold could effectively participate in both biochemical and mechanochemical signaling, as is performed by tissue ECMs. Unlike conventional implantable materials, the scaffold was initially populated by fibroblast-like mesenchymal cells, along with vessel-forming cells, rather than inflammatory mediators. The rapid and robust neovascularization response was consistent with other in vivo studies where oligomer has been implanted into other microenvironments^27,29–33^ and used for in vitro investigations of underlying mechanisms of vessel formation^26,35,59^. As these front-line cells progressed deeper toward the scaffold center with time, tissue neogenesis followed, with formation of adipose tissue and mammary glands, including secretory lobules and ducts. Interestingly, newly formed lobules, which were especially apparent at 4- and 16-week time points, were reminiscent of those found in nulliparous (pre-pregnancy) breasts since they were largely lacking in macrophage infiltration^60^. Collectively, the regenerative tissue response observed with the collagen fill has many similarities to processes associated with tissue development and morphogenesis, including mammary glands^61^, highlighting the importance of maintaining stromal collagen and its associated mechanobiological continuum.

As part of this study, we also documented that the collagen filler was not negatively impacted by radiation therapy and did not compromise interpretation of diagnostic imaging procedures. In the present study, irradiation was applied 2 weeks following simulated lumpectomy, which is within the range of adjuvant radiation administration following BCS. Tumors and tissues with rapid cell turnover, such as the epidermal layer of the skin, are most sensitive to irradiation effects, with the extent of damage depending on the total radiation dose and time over which the radiation is delivered^62^. Irradiation resulted in hyperpigmentation of skin, an expected side effect that is analogous to sunburn or tanning responses displayed in humans, as well as moderate levels of fat necrosis and hyperplasia of glands and ducts. For both collagen and no fill treated groups, the healing progressed similarly to respective non-irradiated groups; however, the healing rate appeared modestly slower based on breast consistency scores and histopathological analysis. Such results were not surprising since irradiation is known to cause delays in wound healing^63^. Based on combined histopathology, x-ray and ultrasound analyses, the collagen filler and its associated signaling capacity were determined to be largely unaffected by irradiation. Radiographs and ultrasonograms also indicated that the collagen fill yielded no suspicious artifacts. This has been a major drawback with fat grafting, where a wide spectrum of alterations in breast tissue have been detected via these diagnostic imaging techniques, ranging from benign-looking lipid cysts to findings suspicious for malignancy such as micro-calcification, focal masses, and speculated areas of increased opacity^64,65^.

Given that this work represented an early proof-of-principle evaluation, these studies are not without limitations. First, owing to breast size differences between pig and human, a quadrantectomy was performed with removal of roughly 25% pig breast volume. Defect volumes ranged from 2 to 5.5 mL, with an average defect volume of about 3 mL. While quadrantectomies are rarely, if ever, performed on women, these absolute defect volumes fell within the range of human clinical procedures. Specifically, published human clinical reports indicate that 67% and 82% of breast tumors are ≤ 1.9 cm (≤ 3.6 mL) and ≤ 2.9 cm (≤ 12.8 mL) in diameter (volume), respectively^66^. While additional studies are needed to determine how defect size affects material performance, no detrimental outcomes are anticipated based on observed material mode of action. However, it is anticipated that time to complete cellularization and healing would vary directly with defect volume. Second, since the longest timepoint evaluated was 16 weeks, additional animal and human clinical studies are needed to define long-term (i.e., 6 months or greater) collagen filler outcomes. A third limitation of these large-animal studies was that pigs were cancer free. As such, the effect of collagen filler on tumor promotion and recurrence cannot be fully evaluated. For a number of reasons, it is not anticipated that the collagen filler would pose a risk to oncologic safety. First, since breast surgeons would be able to more predictably maintain breast contour and consistency, they would have increased confidence about excising more tissue to achieve negative margins. We also show that the collagen filler induces no inflammatory or foreign body response, which is especially important since macrophage infiltration and other processes (e.g., cytokine release) associated with inflammation have been implicated in tumor promotion^67–69^. Additionally, when tested with various cancer cell types in vitro, high fibril density/stiffness of oligomer scaffolds was found to limit tumor cell proliferation and migration^70^. Finally, to further combat tumor recurrence, a chemotherapeutic or other anti-cancer agents could be readily added to the scaffold-forming reaction to achieve targeted and localized delivery. This would dramatically decrease the amount of drug administered and minimize side effects associated with systemic administration.

In conclusion, our work shows that a regenerative tissue filler that forms in situ and is fashioned from a natural collagen polymer appears to address surgeon needs and overcome major limitations associated with conventional implantable materials. To the best of our knowledge, this is the first report of a breast filler that persists, maintains its volume, and induces progressive breast tissue regeneration, including mammary glands, ducts, and adipose tissue. Additionally, study findings have important implications to regenerative medicine, suggesting that decreased inflammation and maintenance of a collagen structural and mechanical continuum tilts the healing balance from repair (scar formation) towards regeneration. This work sets the stage for future pre-clinical and clinical studies where the translation potential of this prototype regenerative tissue filler can be further validated for BCS and other soft tissue restoration and reconstruction needs.

## Methods

### Scaffold-forming Type I Collagen

The scaffold-forming collagen was obtained as a kit from GeniPhys (Zionsville, Indiana) as shown if Fig. 1a. The oligomeric collagen component of these kits was manufactured and quality-controlled from hides (dermis) of closed herd pigs in accordance with patented procedures and ASTM International F3089-14 guidelines for polymerizable collagens^71,72^. Two collagen formulations that differed by a single, proprietary manufacturing step were evaluated; however, since no difference in performance was observed, results were combined and presented as a single formulation. To evaluate collagen purity, sodium dodecyl sulfate polyacrylamide gel electrophoresis (SDS–PAGE) was performed on collagen samples and molecular weight standards (Novex SeeBlue Plus2, Invitrogen, Carlsbad, CA) using 4–20% and 6% gels (Invitrogen) and stained with Coomassie Blue (Sigma-Aldrich, St. Louis, MO) according to established methods^25^. Collagen concentration was determined using a Sirius Red (Direct Red 80, Sigma-Aldrich) assay. Time-dependent oscillatory shear rheometry was performed to determine self-assembly kinetics and shear storage (G′) and loss (G″) moduli. Briefly, neutralized oligomeric collagen samples were tested on an AR2000 rheometer (TA Instruments, New Castle, DE), with a 40-mm parallel plate geometry and solvent trap. Prior to sample loading and during the first 2 min of testing, the Peltier plate was maintained at 4 °C. Oscillatory shear measurements were taken at 1% strain for this initial 2 min and continued for 10 min after the temperature was increased to 37 °C. Following oscillatory shear testing, the sample was subjected to unconfined compression testing at a strain rate of 20 µm/s. To define the kinetics of scaffold formation, a plot of shear storage modulus over time was created, and the time at which the collagen reached its maximum stiffness (G′) was defined as the polymerization time. This point was also used to define scaffold G′ and G″ values. To obtain the compression modulus, stress–strain curves were created from the unconfined compression data and the slope was calculated in a specified low strain region (20–40% strain), corresponding to the low stress/strain moduli that are reported in literature for human breast tissue^54,73^. Four independent batches of prototype collagens were tested with 6–8 replicates per batch (N = 4 batches; n = 6–8 replicates per batch).

### Porcine simulated lumpectomy model

Simulated lumpectomies were performed on female Yucatan mini-pigs (retired breeders) weighing between 45 and 65 kg using a protocol that was approved by the Purdue Animal Care and Use Committee. All handling and care of animals was performed in accordance with relevant NIH and AAALAC guidelines. Prior to surgery, all of the breasts of an individual pig (12 breasts per pig) were randomly assigned to experimental (6 breasts per pig) and control groups, with no fill (3 breasts per pig) and no surgery (3 breasts per pig) serving as negative and positive controls, respectively. Animals were anesthetized, intubated, and placed in dorsal recumbency. For each simulated lumpectomy, a 3-cm skin incision was made using a scalpel, with incisions oriented transversely and placed immediately lateral to the nipple-areolar complex of each breast. Approximately one quarter of the mammary tissue was excised using electrocautery and its volume measured using a standard volume displacement method. A subset of excised normal mammary tissue was subjected to unconfined compression testing (strain rate: 1 mm/min, compression modulus determined in linear region of 20–40% strain) for characterization of mechanical properties. Titanium marker clips (Ethicon Small LIGACLIP, West CMR, Clearwater, FL) were placed in a subset of animals to facilitate margin identification of collagen and no fill treated surgical sites. For collagen-treated sites, the collagen solution and neutralization reagent were mixed according to instructions, and the resultant neutralized liquid collagen used to fill the surgical void. Negative control sites received no fill (untreated). A subset of pig breasts that were not subjected to surgery served as positive controls. All surgical sites were closed using resorbable sutures and bandaged with a non-adherent pad (McKesson, San Francisco, CA) and Tegaderm (3 M, St. Paul, MN) dressing. The animals’ health status was monitored daily based on appetite, attitude, movement, and elimination.

#### Longitudinal study

A longitudinal study was performed with post-surgical assessment performed at 1-, 4-, and 16-week time points (2 animals per time point) to achieve twelve replicates (n = 12) for the collagen filler group and six replicates (n = 6) for each the no fill and no surgery control groups.

#### Radiation study

To address the question of how radiation therapy affects the tissue response to collagen soft-tissue fillers, two animals were treated with radiation following simulated lumpectomy and treatment. Pig breasts were again randomly assigned to treatment groups, with no fill treatment and breasts on which no surgery was performed serving as negative and positive controls, respectively. Computed tomography (CT) based, three-dimensional conformal treatment (3D-CRT) plans were generated for each animal. Two weeks following surgery, a 6 MV Varian EX linear accelerator and 120-leaf multi-leaf collimator was used to apply 4 Gy radiation to the ventral surface daily for 5 days for a total dose of 20 Gy. Post-surgical assessments were performed 4 and 16 weeks following surgery with six replicates (n = 6) for the collagen filler group, three replicates (n = 3) for the no fill group, and one (n = 1) replicate for the no surgery group. The most caudal pair of mammary glands served as non-irradiated no surgery controls. Outcomes from irradiated animals were compared to non-irradiated animals from the longitudinal study.

### Post-surgical procedures and assessment

At designated time points, the animals were anesthetized, and each breast evaluated using a semi-quantitative scoring system for gross breast/surgical site appearance, including erythema/eschar formation and edema formation, and breast uniformity/consistency scoring as shown in Supplementary Fig. S3. Additionally, ultrasound imaging of each mammary gland was performed with a Mindray M7 ultrasound machine (Mindray North America, Mahwah, NJ) and a linear 4–7 MHz transducer. Following euthanasia, a mastectomy was performed on each breast, maintaining all surgical sites, any implant, and the surrounding tissue. Each breast was placed in 10% buffered formalin and radiographed using an InnoVet Select Radiograph unit (Summit, Niles, IL) with a Genesis Vet DR plate installed using Genesis VxVue acquisition software (Genesis Digital Imaging, Los Angeles, CA), prior to processing for histopathological analysis. Formalin-fixed explanted tissues were bisected and imaged prior to paraffin embedding and sectioning. Sections were stained with hematoxylin and eosin (H&E). To detect epithelial cells, sections were stained for pan cytokeratin (ab9377, Abcam, Cambridge, MA) at a dilution of 1:100 and then treated with secondary DyLight 488 goat anti-rabbit (DI-1488, Vector Labs, Burlingame, CA) at 6 μg/mL. Nuclei were counterstained with DAPI (4′, 6-diamidino-2′-phenylindole, dihydrochloride; EN62248, Pierce Biotechnology, Rockford, IL). Images were acquired using a Aperio VERSA 8 whole-slide scanner (Leica Biosystems, Buffalo Grove, IL).

## Data availability

All data are included in this paper or the Supplementary Materials. Additional materials can be obtained by request to T.J.P. and S.V.-H. and may be subject to non-disclosure and material-transfer agreement requirement with GeniPhys.

## Supplementary Information

Supplementary Video 1.

Supplementary Information 1.
