# Supplementary material for: Regenerative tissue filler for breast conserving surgery and other soft tissue restoration and reconstruction needs
Source: Sci Rep. 2021 Feb 1;11:2711. doi: 10.1038/s41598-021-81771-x (PMC7851166; doi:10.1038/s41598-021-81771-x)
Supplement: Supplementary file 2 — Supplementary Information 1. [file 41598_2021_81771_MOESM2_ESM.docx]

**SUPPLEMENTARY INFORMATION**

Regenerative tissue filler for breast conserving surgery and other soft tissue restoration and reconstruction needs

**Authors:** Theodore J. Puls^1^, Carla S. Fisher^2^, Abigail Cox^3^, Jeannie M. Plantenga^4^, Emma L. McBride^5,6^, Jennifer L. Anderson^5^, Craig J. Goergen^5^, Melissa Bible^5^, Tracy Moller^5^, Sherry L. Voytik-Harbin^5,7^*

**Affiliations:**

^1^GeniPhys, LLC, Zionsville, Indiana 46077.

^2^Division of Surgery, Indiana University School of Medicine, Indianapolis, IN 46202.

^3^Department of Comparative Pathobiology, College of Veterinary Medicine, Purdue University, West Lafayette, IN 47907.

^4^Department of Veterinary Clinical Sciences, College of Veterinary Medicine, Purdue University, West Lafayette, IN 47907.

^5^Weldon School of Biomedical Engineering, College of Engineering, Purdue University, West Lafayette, IN 47907.

^6^Medical Scientist/Engineer Training Program, Indiana University School of Medicine, Indianapolis, IN 46202.

^7^Department of Basic Medical Sciences, College of Veterinary Medicine, Purdue University, West Lafayette, IN 47907.

*****Corresponding author: S. L. Voytik-Harbin. email: harbins@purdue.edu

**Supplementary Video S1. Prototype collagen filler exhibits in situ scaffold formation.** Type I oligomeric collagen rapidly transitions from its initial liquid form to a stable, shape-retaining fibrillar collagen scaffold following injection into a contoured geometry maintained at body temperature (37°C). Scaffold-forming reaction is shown in real time. (Video can be viewed online.)

**
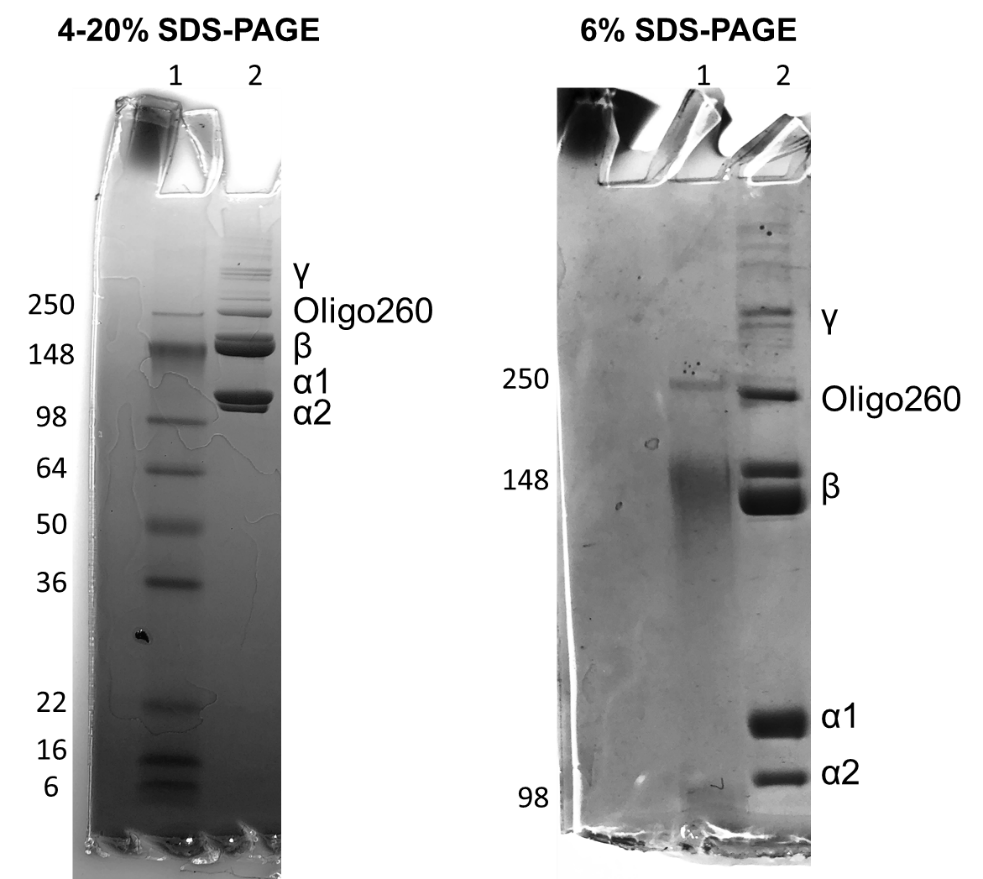
**

**Supplementary Figure S1. Full Length SDS-PAGE gels.** SDS-PAGE (4-20% and 6% gels) documenting purity and characteristic banding pattern of type I oligomeric collagen. Images represent full length gels (including the edges of the gel) and show all relevant lanes for this manuscript. Lane 1: molecular weight standard. Lane 2: type I oligomeric collagen.


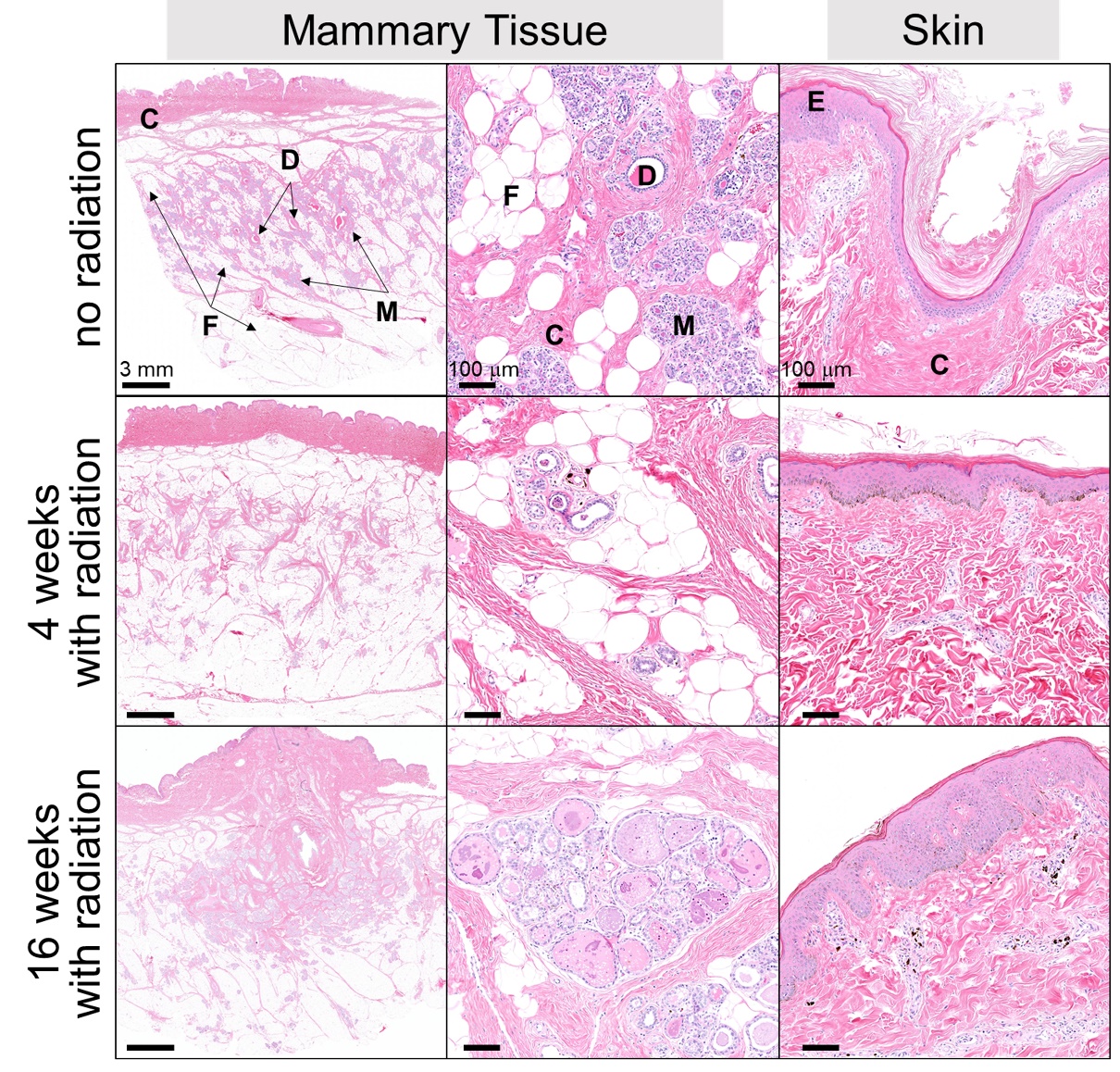


**Supplementary Figure S2.** **Multiple tissue type composition of normal breast tissue and overlying skin and effects of irradiation.** Cross-sections (H&E) of normal breast tissue and associated skin from pigs receiving no irradiation and 4 weeks and 16 weeks after lumpectomy with radiation. Mammary tissue is composed of collagenous connective tissue (C), mammary gland lobules (M), mammary ducts (D), and adipose (fatty) tissue (F). Skin contains a multi-cellular epidermal layer (E) with an underlying collagenous dermis (C).


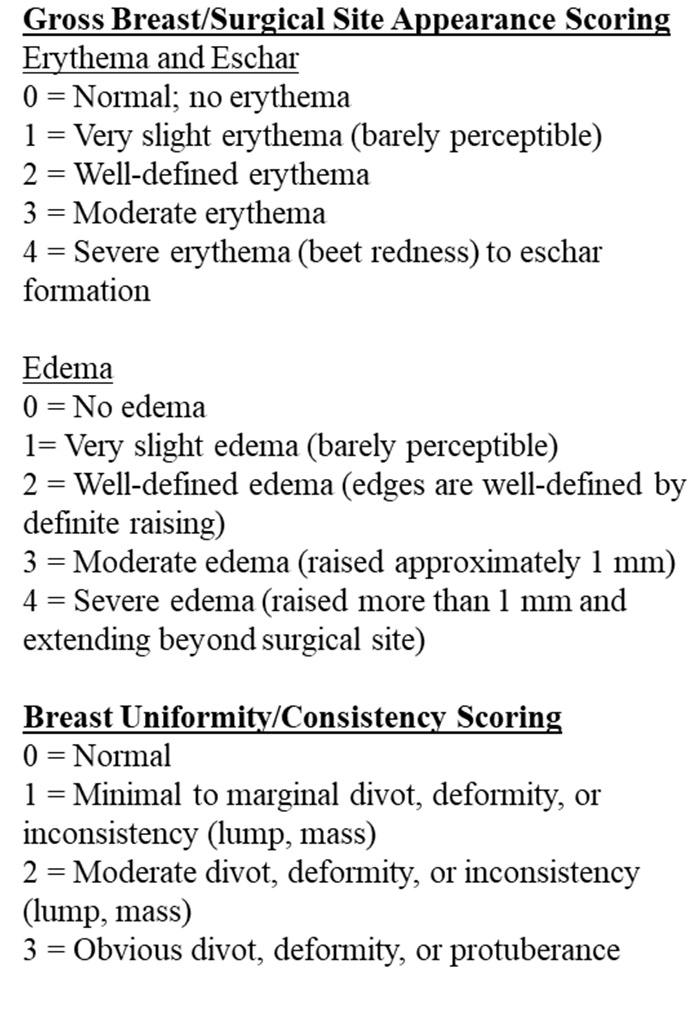


**Supplementary Figure S3. Semi-quantitative scoring used for post-surgical assessment of pig breasts.** Breasts and surgical sites were assessed based on gross appearance, including evidence of erythema and eschar as well as edema. Palpation was used to assess breast uniformity and consistency.


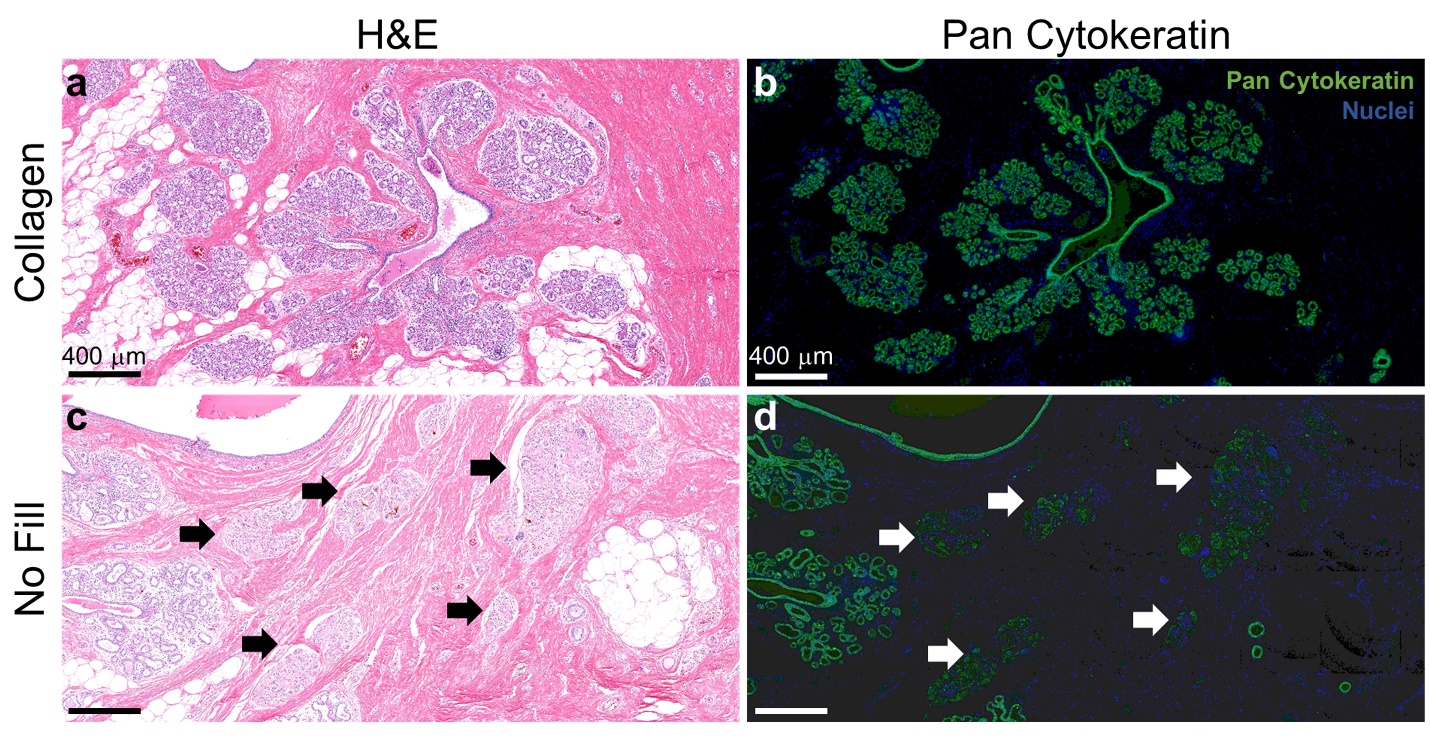


**Supplementary Figure S4. Collagen filler supports mature and robust mammary gland morphogenesis, while no fill voids show altered gland architecture with inflammatory cells.** Corresponding **(a,c)** H&E and **(b,d)** pan cytokeratin stained cross-sections of surgical voids 16 weeks following treatment with the **(a,b)** collagen filler or **(c,d)** no fill, with selected regions representing the periphery of the collagen filler and formed scar tissue, respectively. Pan cytokeratin highlights epithelial cells lining mammary lobules and ducts within collagen fill and no fill groups. Collagen fill group shows mature and robust glandular architecture, while numerous glands with altered morphology and few inflammatory cells (black and white arrows) were identifiable in the no fill group. Immunofluorescence images shows pan cytokeratin (green), with nuclei counterstained with DAPI (blue).
